# Biological mechanisms associated with increased perseveration and hyperactivity in a genetic mouse model of neurodevelopmental disorder

**DOI:** 10.1016/j.psyneuen.2012.12.002

**Published:** 2013-08

**Authors:** Simon Trent, Rachel Dean, Bonnie Veit, Tommaso Cassano, Gaurav Bedse, Obah A. Ojarikre, Trevor Humby, William Davies

**Affiliations:** a Behavioural Genetics Group and Neuroscience and Mental Health Research Institute, Schools of Psychology and Medicine, Cardiff University, Cardiff, UK; b Institute of Psychological Medicine and Clinical Neurosciences and MRC Centre for Neuropsychiatric Genetics and Genomics, School of Medicine, Cardiff University, Cardiff, UK; c School of Psychology, Cardiff University, Cardiff, UK; d Department of Clinical and Experimental Medicine, Medical School, University of Foggia, Foggia, Italy; e Department of Physiology and Pharmacology, Sapienza University of Rome, Rome, Italy; f Division of Stem Cell Biology and Developmental Genetics, MRC National Institute for Medical Research, London, UK

**Keywords:** Acetylserotonin O-methyltransferase, Attention deficit hyperactivity disorder, Autism, Dehydroepiandrosterone, Hippocampus, Locomotor activity, Serotonin, Spatial working memory, Steroid sulfatase, Xp22.3

## Abstract

Chromosomal deletions at Xp22.3 appear to influence vulnerability to the neurodevelopmental disorders attention deficit hyperactivity disorder (ADHD) and autism. 39,X^Y*^O mice, which lack the murine orthologue of the Xp22.3 ADHD candidate gene *STS* (encoding steroid sulfatase), exhibit behavioural phenotypes relevant to such disorders (e.g. hyperactivity), elevated hippocampal serotonin (5-HT) levels, and reduced serum levels of dehydroepiandrosterone (DHEA). Here we initially show that 39,X^Y*^O mice are also deficient for the recently-characterised murine orthologue of the Xp22.3 autism candidate gene *ASMT* (encoding acetylserotonin-O-methyltransferase). Subsequently, to specify potential behavioural correlates of elevated hippocampal 5-HT arising due to the genetic lesion, we compared 39,X^Y*^O MF1 mice to 40,XY MF1 mice on behavioural tasks taxing hippocampal and/or 5-HT function (a ‘foraging’ task, an object-location task, and the 1-choice serial reaction time task of impulsivity). Although *Sts*/*Asmt* deficiency did not influence foraging behaviour, reactivity to familiar objects in novel locations, or ‘ability to wait’, it did result in markedly increased response rates; these rates correlated with hippocampal 5-HT levels and are likely to index behavioural perseveration, a frequent feature of neurodevelopmental disorders. Additionally, we show that whilst there was no systematic relationship between serum DHEA levels and hippocampal 5-HT levels across 39,X^Y*^O and 40,XY mice, there was a significant inverse linear correlation between serum DHEA levels and activity. Our data suggest that deficiency for genes within Xp22.3 could influence core behavioural features of neurodevelopmental disorders via dissociable effects on hippocampal neurochemistry and steroid hormone levels, and that the mediating neurobiological mechanisms may be investigated in the 39,X^Y*^O model.

## 1 Introduction

Potentially pathogenic cytogenetic deletions within Xp22.3 are relatively common in patients with autistic spectrum disorders (ASDs) (Thomas et al., 1999; Chocholska et al., 2006; Vorstman et al., 2006; Kent et al., 2008; Shinawi et al., 2009), characterised by social/communication impairments, restricted interests or repetitive/stereotyped behaviours, hyperactivity and anxiety (O’Hare, 2009). Xp22.3 deletions have also been reported in cases of attention deficit hyperactivity disorder (ADHD) (Boycott et al., 2003; Lonardo et al., 2007; Kent et al., 2008), a second neurodevelopmental condition characterised by inattention, pathological impulsivity and hyperactivity (Thapar et al., 2012) which is commonly comorbid with autism and which is thought to share overlapping genetic aetiology (Rommelse et al., 2010); individuals with ADHD may also display a heightened tendency towards behavioural perseveration (Fischer et al., 2005; Tsuchiya et al., 2005).

Several Xp22.3 genes represent good candidates for conferring vulnerability to psychiatric illness: functional mutations within *NLGN4X* (encoding the synaptic neuroligin 4X protein) have been reported in cases of autism (Jamain et al., 2003; Laumonnier et al., 2004), whilst mutations within *ASMT* (encoding the acetylserotonin-O-methyltransferase enzyme involved in the biosynthesis of melatonin from serotonin) have been seen in individuals with autism (Melke et al., 2008; Jonsson et al., 2010) and ADHD (Chaste et al., 2011). Cytogenetic deletions encompassing *STS* (encoding the enzyme steroid sulfatase (Sts)) and inactivating mutations/genetic variants within the gene can influence ADHD risk and presentation (Kent et al., 2008; Stergiakouli et al., 2011). Sts cleaves sulfate groups from various steroid hormones, thereby altering their potency and specificity at a variety of neurotransmitter receptors (Reed et al., 2005). Besides putative effects on neurodevelopmental disorders, Sts deficiency has recently been implicated in postpartum psychosis risk (Davies, 2012). Understanding how lack of the gene products above impacts upon neurobiology and behaviour will be important for illuminating the pathophysiological basis of multiple psychiatric conditions.

39,X^Y*^O mice, which possess a single sex chromosome composed of an X and Y chromosome fused at the pseudoautosomal region (PAR) (Odorisio et al., 1998), exhibit behavioural and endocrinological phenotypes of relevance to ADHD and ASDs including inattention (Davies et al., 2009), hyperactivity, heightened emotional reactivity, occasional aggression, and reduced systemic levels of a major Sts product, dehydroepiandrosterone (DHEA) (Trent et al., 2012b). Analysis of whole-tissue neurochemical levels across the brain in 39,X^Y*^O and 40,XY mice indicated reduced levels of the noradrenaline metabolite 3-methoxy-4-hydroxyphenylglycol (MOPEG) in the striatum and elevated levels of striatal and hippocampal serotonin (5-hydroxytrypamine, 5-HT) in the former group. The hippocampal 5-HT finding was highly significant, and there appeared to be a particularly strong correlation between hippocampal 5-HT levels and aspects of behavioural function (Trent et al., 2012a). Until recently, *Sts* was the only known gene within the mouse PAR and thus represented the sole candidate for the above phenotypes. However, emerging genetic data (Kasahara et al., 2010 and www.ncbi.nlm.nih.gov/projects/mapview) have suggested that the *Asmt* and *Erdr1* (erythroid differentiation regulator 1) genes, together with the poorly annotated *LOC100861696*, and *G530011O06Rik* genes, may also be pseudoautosomal; the chromosomal locations of the former two have recently been changed from ‘unknown’ to sex-linked in the Mouse Genome Informatics database (www.informatics.jax.org, updated October 2012). Hence, we initially examined the extent of the deleted region in the 39,X^Y*^O model to ascertain whether the absence of genes other than *Sts* could contribute to its phenotype.

Given our previous data suggesting altered hippocampal neurochemistry in *Sts*-deficient 39,X^Y*^O mice (Trent et al., 2012a), and the observation that acute Sts inhibition can result in altered hippocampal neurochemistry (Rhodes et al., 1997), we next tested 39,X^Y*^O mice on two behavioural tasks sensitive to hippocampal function to specify potential behavioural correlates of these neurochemical changes: (i) an ethobiologically-relevant spatial navigation (‘foraging’) task conceptually analogous to ‘open field’ tasks used in rodents and pigeons (Hermes et al., 2005; Pearce et al., 2005), in which subjects are required to visit and consume reinforcer from baited pots in the most parsimonious manner possible and (ii) an ‘object-location task’, in which the relative exploration times of objects in a sample spatial context, and of the same ‘familiar’ objects in a revised spatial context, are compared (Good et al., 2007). Being land-based and appetitively-motivated (in the case of the foraging task), neither task should be significantly confounded by factors such as anxiety and swimming ability inherent in other commonly-used tasks of hippocampal function such as the Morris Water Maze; this was particularly important given evidence for heightened emotionality in 39,X^Y*^O mice (Trent et al., 2012b).

There is prior evidence that reducing hippocampal 5-HT levels via lesion of the median raphe nucleus in rats increases premature responding (i.e. decreases ‘ability to wait’) in the 5-choice serial reaction time task (5-CSRTT) (Harrison et al., 1997). We have previously shown that 39,X^Y*^O mice show a significant reduction in premature responding in the 5-CSRTT (Davies et al., 2009). Here, we compared the performance of 39,X^Y*^O and wildtype mice on a modified version of the 5-CSRTT in which the stimulus was only presented at a central location (1-choice SRTT) to assay ‘ability to wait’ in the absence of any confounding effects on visuospatial attention; we then measured hippocampal 5-HT levels in these mice. We hypothesised that 39,X^Y*^O mice would exhibit reduced premature responding relative to wildtype mice, and that hippocampal 5-HT levels would be inversely correlated with levels of premature responding.

We previously proposed that reduced DHEA levels in 39,X^Y*^O mice might result in elevated hippocampal 5-HT levels by increasing neural tryptophan hydroxylase activity (Trent et al., 2012a). Here, we indirectly tested this idea by assaying DHEA levels and hippocampal 5-HT levels within the same animals, predicting an inverse relationship between the two variables. Finally, in humans, there is preliminary data suggesting that blood levels of DHEA in subjects diagnosed with ADHD are inversely correlated with symptom severity, notably measures of hyperactivity (Strous et al., 2001; Wang et al., 2011). Our experimental set-up allowed us to explicitly test for a relationship between serum DHEA levels and locomotor activity in the absence of confounds associated with testing in clinical populations such as previous or current pharmacotherapy.

## 2 Methods

### 2.1 Subjects and animal husbandry

39,X^Y*^O and 40,XY mice were bred and genotyped at the MRC National Institute for Medical Research, London, UK as described previously (Davies et al., 2009; Trent et al., 2012a,b), before being transferred to Cardiff University for behavioural testing. In Cardiff, subjects were treated with Baytril and Norodine-24 antibiotics for one month in a negative-pressure isolator to cure a *Pasteurella pneumotropica* infection prior to release onto the open racks. The mice were then housed in a holding room (1–4 mice of the same karyotype per cage) maintained at 21 ± 2 °C and 50 ± 10% humidity, with a 12-h light–dark cycle (lights on at 0700 h). Single-housing of some 39,X^Y*^O mice was necessary to prevent fighting, and did not appear to impact significantly on the pertinent measures assayed here (data not shown). Behavioural testing was motivated where necessary by using a 10% condensed milk reinforcer (Nestle Ltd.), previously shown to be highly palatable (>70% preference over tap water) and equivalently preferred by 39,X^Y*^O and 40,XY mice (Davies et al., 2009; Trent et al., 2012a); prior to behavioural testing, mice were subject to a water restriction schedule, whereby they received 4 h of water per day for four days, and 2 h/day thereafter. All experiments were conducted according to the Animal (Scientific Procedures) Act 1986. All efforts were made to minimise animal suffering, to reduce the number of animals used, and to utilise alternatives to in vivo techniques, if available.

### 2.2 Genetic analysis

Genomic DNA was obtained from 40,XY (*n* = 2) and 39,X^Y*^O (*n* = 2) brain tissue as described in Davies et al. (2009). *Asmt*, *Erdr1*, *Mid1* and *Sts* genes were amplified by PCR in separate mixes: 18.25 μl sterile water, 2.5 μl 10× buffer, 1.0 μl 5 mM dNTPs, 200 nM forward and reverse primers, 0.25 μl HotStar Taq polymerase, 1.0 μl DNA solution under the following conditions: 94 °C for 15 min [94 °C for 40 s, 60 °C for 40 s, 72 °C for 1 min 30 s, for 40 cycles], 72 °C for 10 min. Primers used were as follows: AsmtF: 5′-AGGAGACCTGGAGCCTGTG-3′, AsmtR: 5′-GTCTCGAACACGGTGACCTC-3′, Erdr1F: 5′-TAGCCGCAGCTATGGTTTCT-3′, Erdr1R: 5′-CAGGCTTCCTACCTTGTGGA-3′, Mid1F: 5′-AGCCTGTGGAGTCCATCAAC-3′, Mid1R: 5′-GCTTTCAGGCACTCATCACA-3′, StsF: 5′-GCTCGCTGACATCATCCTC-3′, StsR: 5′-CACCGATGCCCAGGTCGTC-3′. Products were visualised on a 1.5% ethidium bromide-stained agarose gel.

### 2.3 Foraging task

39,X^Y*^O (*n* = 12) and 40,XY (*n* = 12) mice (8–10 months) were run in a pseudorandom order between 1000 and 1500 h. After shaping and habituation procedures, mice were given a series of test and probe sessions (see Supplementary Material). During five consecutive test sessions, mice were required to collect drops of 10% condensed milk reinforcer from eight pots; these sessions lasted until all reinforcers had been collected, or for a maximum of 15 min. Main measures of interest at baseline performance (i.e. test day 5) included the number of reinforcers obtained within the time limit, the latency to obtain these reinforcers, and the number of errors made in obtaining the reinforcers (i.e. repeat visits to previously visited containers). The two subsequent probe sessions were designed to test for any differential group effects on the use of extra-maze cues, and on perseverative behaviour (as indexed by repeat visits to a single previously-visited pot).

### 2.4 Object-location task

A randomly-selected subset of 39,X^Y*^O (*n* = 8) and 40,XY (*n* = 8) mice who had completed the foraging task were run on this task between 1000 and 1500 h; these mice were allowed ad libitum access to homecage food and water. Following habituation to the test arena and the test objects, subjects were given two test sessions, each composed of a sample and test phase two minutes apart. In the sample phase, mice were allowed to explore four objects located in a square in the centre of the arena for a maximum of 10 min (with exploration times equalised across karyotypes); in the test phase, the two diagonally-opposed objects were switched, and mice were allowed to explore the novel configuration for 10 min. For test days one and two, the opposite combination of objects was switched. Data were averaged across test days one and two, with the main measure of interest being the change in exploration of the ‘switched objects’ in their novel configuration (test phase) relative to their exploration in their initial configuration (sample phase). Further experimental details are available in the Supplementary Material.

### 2.5 1-Choice serial reaction time task (1-CSRTT)

Testing was performed in 9-hole boxes (Campden Instruments, UK)(Humby et al., 1999) between 1000 and 1500 h. 39,X^Y*^O (*n* = 12) and 40,XY (*n* = 11) mice aged 8–10 months were trained to make a rapid nosepoke response to a light stimulus presented in the central hole in order to obtain 20 μl of 10% condensed milk reinforcer (Nestle Ltd.). Initial shaping was as described previously for the 5-CSRTT (Humby et al., 1999) with one session per day, with the stimulus duration being reduced in a stepwise manner from 32 s to 1 s at baseline once criteria had been met for two consecutive sessions (>50 trials, >80% accuracy, <30% omissions). At baseline, the latency between trial initiation by panel push and stimulus onset (i.e. the inter-trial interval, ITI) was fixed at 5 s. Once stable baseline performance had been achieved (using the aforementioned criteria), subjects were given three manipulation sessions, with at least one stable baseline session between each. In the first manipulation session (LITI), the ITI was pseudorandomly varied between 5, 6, 7 and 8 s. In the second manipulation session (ELITI), the ITI was pseudorandomly varied between 5, 7, 9 and 11 s. In the final manipulation session (I10), the ITI was held constant at 10 s. The main measures of interest were: number of trials, accuracy, % omissions, number of premature nosepokes within ITI (main index of ‘ability to wait’), total number of nosepokes, number of panel pushes to initiate trials, and activity (indexed by infra-red beam breaks).

### 2.6 Locomotor activity

Locomotor activity was assayed in mice on ad libitum food and water who managed to acquire and complete the 1-CSRTT (39,X^Y*^O: *n* = 12, and 40,XY: *n* = 6). Testing was conducted in clear perspex cages (21 cm × 36 cm × 20 cm, *l* × *b* × *h*) fitted with an infrared beam 3 cm from either end and 1 cm from the floor. Mice were allowed to explore freely for 3 h; for the first hour the test was run under dim lighting (20 lx) and for the remaining time it was run in the dark; the onset of the dark period coincided with the onset of the dark period in the holding room. The main measure of activity was the number of beam breaks throughout the test period.

### 2.7 Brain dissection and high performance liquid chromatography (HPLC) for determination of 5-HT levels

Mice (aged 10–12 months) that had undergone testing on the 1-CSRTT and locomotor activity paradigms were allowed ad libitum access to water and food prior to being culled by cervical dislocation. Brains were removed immediately and the entire hippocampus was dissected from the right hemisphere and weighed using a microbalance before being frozen on dry ice and stored at −80 °C. Samples were processed and analysed for wet weight-normalised monoamine levels as described previously (Trent et al., 2012a).

### 2.8 DHEA analysis

Trunk blood was collected between 1000 and 1500 h from 39,X^Y*^O (*n* = 18) and 40,XY (*n* = 13) mice on ad libitum food and water, including the subset used for hippocampal dissections. Serum was extracted, and analysed for DHEA levels by enzyme-linked immunosorbent assay (ELISA) as described in Trent et al., 2012b. Sample outliers (i.e. values >2 standard deviations below or above the group mean) were excluded from the analysis.

### 2.9 Statistics

Data were analysed using SPSS 18.0 (IBM Corporation, New York). Data were tested for normality using the Shapiro–Wilks test. Normal data were analysed by unpaired two-tailed *t*-test/one-way ANOVA/two-way repeated measures ANOVA, with factors of KARYOTYPE and TIMEPOINT. Skewed data were transformed before parametric analysis as appropriate. Where sphericity assumptions were violated in two-way ANOVA, Greenhouse–Geisser corrected degrees of freedom values are presented. Non-parametric data were analysed by two-tailed Mann–Whitney *U* test. For the correlational analyses, Pearson's test was used for normally distributed data, and Spearman's test for non-normally distributed data. Frequency data were analysed by chi-squared test, or Fisher Exact Test if cells contained fewer than five values. *p*-Values of <0.05 were regarded as significant. Data are presented as mean values ± standard error of the mean.

## 3 Results

### 3.1 Specification of the genetic lesion in 39,X^Y*^O mice

**Figure 1:**
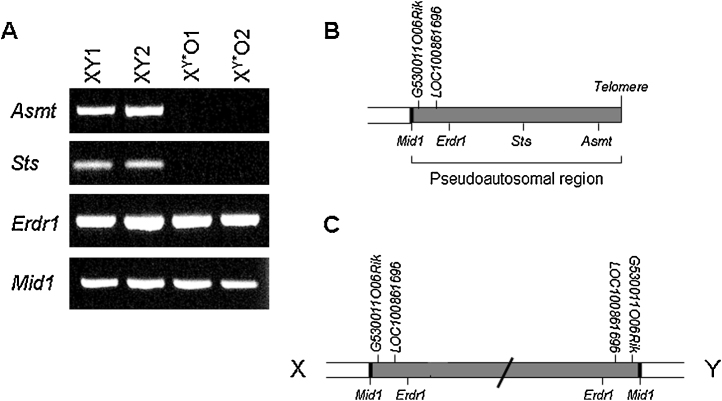
Presence of putative pseudoautosomal genes in 40,XY (*n* = 2) and 39,X^Y*^O (*n* = 2) mice as indexed by PCR from genomic DNA (A); *Asmt* and *Sts* genes are deleted in the latter group, whilst the *Erdr1* and *Mid1* genes (and therefore *G530011O06Rik* and *LOC100861696* genes) are retained. These data indicate that the pseudoautosomal regions of the X and Y chromosomes (B) form an end-to-end fusion at the region between *Erdr1* and *Sts* in 39,X^Y*^O mice (C).

PCR analysis indicated that the genes *Sts* and *Asmt* are both deleted in the 39,X^Y*^O mouse, but that neither *Mid1* (spanning the pseudoautosomal boundary) nor *Erdr1* is (Fig. 1A); *LOC100861696* and *G530011O06Rik* which are located between *Mid1* and *Erdr1* (Fig. 1B) will also be retained in 39,X^Y*^O mice. These data help to refine the genetic lesion in 39,X^Y*^O mice, indicating that the X–Y fusion occurs somewhere between the *Erdr1* and *Sts* genes (Fig. 1C).

### 3.2 Hippocampal 5-HT levels

5-HT levels in one 39,X^Y*^O sample could not be reliably determined. Consistent with our previous data (Trent et al., 2012a), in this separate experimental cohort, we observed a highly significant increase in hippocampal 5-HT levels in 39,X^Y*^O tissue relative to 40,XY (529 ± 29 pg/mg vs. 340 ± 40 pg/mg respectively, *t*[15] = −3.835, *p* = 0.002). 5-HT turnover (as indexed by the ratio of 5-hydroxyindolacetic acid:5-HT levels) was again also found to be significantly reduced (by ∼26%) in the hippocampus of 39,X^Y*^O mice relative to 40,XY mice (data not shown).

### 3.3 ‘Foraging’ task

At baseline (i.e. test day 5), there was no between-group difference in the number of mice collecting reinforcer from all eight containers within the 15 min time limit (7 40,XY mice, 10 39,X^Y*^O mice, two-tailed Fisher Exact Test, *p* = 0.37); 39,X^Y*^O mice which completed the task within the time limit did so significantly more rapidly than those 40,XY mice which did (326 ± 42 s vs. 593 ± 92 s, *t*[15] = 2.914, *p* = 0.011); this faster completion latency is more likely to reflect hyperactivity in the former group than enhanced cognition as 39,X^Y*^O mice made significantly more quadrant crosses during the initial arena habituation than 40,XY mice (84 ± 7 vs. 59 ± 4, *t*[15] = −2.691, *p* = 0.017). 39,X^Y*^O and 40,XY mice which completed the task did not differ in their total errors (11 ± 2 vs. 13 ± 2 respectively, *t*[15] = 0.546, *p* = 0.593); as expected, the majority of these errors were made during the second half of the task (i.e. following consumption from the fourth container) when the likelihood of correctly identifying a reinforced container by chance was reduced (39,X^Y*^O: 10 ± 2 and 40,XY: 11 ± 3). The fact that both groups of mice made such errors suggests that neither were using odour cues to any great extent, or that neither were using such cues effectively; furthermore, we did not detect any evidence that mice were using kinaesthetic mediating strategies such as consistently visiting pots in a clockwise direction to complete the task. Hence, the task might reasonably be regarded as taxing ‘spatial working memory’. Equally low levels of defecation were seen in both groups of mice (39,X^Y*^O: 0.4 ± 0.3 faecal boli, 40,XY: 0.7 ± 0.3 faecal boli, *p* = 0.410) consistent with generally low levels of emotional reactivity within the task across both groups.

In the absence of extra-maze cues, there was no between-group difference in the number of mice collecting reinforcer from all eight containers within the 15 min time limit (9 40,XY mice, 11 39,X^Y*^O mice, two-tailed Fisher Exact Test, *p* = 0.59). Mutant and wildtype mice completed this variant of the task with equivalent latencies (386 ± 62 s and 447 ± 53 s respectively, *t*[18] = 0.727, *p* = 0.476). As predicted, mice completing the task tended to make more errors in the absence of extra-maze cues than in their presence, but again, there was no significant effect of KARYOTYPE (39,X^Y*^O: 12 ± 3, 40,XY: 14 ± 4, *p* = 0.603). These data suggest that whilst both groups of mice were using extra-maze cues to guide their behaviour to some extent, they were equivalently more reliant upon intra-maze and/or kinaesthetic cues to complete the task.

11 39,X^Y*^O and 11 40,XY mice consumed the reinforcer within the 10 min maximum test period in the one-pot task variant (*p* = 1.0). 39,X^Y*^O mice were significantly faster to consume the one sample than 40,XY mice (70 ± 20 s vs. 127 ± 18 s respectively, *t*[20] = 2.126, *p* = 0.046), and made more than twice the number of repeat visits to the pot once the reinforcer had been consumed (15 ± 2 vs. 7 ± 1 respectively, *t*[20] = −3.647, *p* = 0.002); the number of repeat visits in the 39,X^Y*^O group remained significantly increased even after normalising for the fact that these mice had a significantly longer time interval in which to make such visits (100× repeat visits/s: 2.8 ± 0.4 s^−1^ vs. 1.4 ± 0.2 s^−1^ respectively, *t*[20] = −3.586, *p* = 0.002). There was a highly significant positive correlation between the number of times an animal re-visited a pot in which the reinforcer had already been consumed and its activity in the ‘open field’ session (*r* = 0.633, *p* = 0.002).

### 3.4 Object-location task

During the 10 min sample phase, total investigation time (all four objects) was 62 ± 5 s across both groups, and as in the foraging task, emotional reactivity as indexed by faecal boli number was equally low across both groups (39,X^Y*^O: 2.8 ± 1.1, 40,XY: 1.8 ± 0.7, *p* = 0.798). 39,X^Y*^O and 40,XY groups spent approximately half of their time exploring the two objects to be swapped in the test phase (50 ± 2% vs. 50 ± 2% respectively, *t*[14] = 0.246, *p* = 0.809). During the 10 min test phase, both 39,X^Y*^O and 40,XY groups spent more time investigating all four objects than in the sample phase, but again there was no effect of KARYOTYPE on this measure (39,X^Y*^O: 71 ± 9 s, 40,XY: 91 ± 7 s, *p* = 0.074). At test, both groups showed equivalent, and >50%, exploration of the two objects that had been swapped (39,X^Y*^O: 59 ± 3%, 40,XY: 62 ± 4%, *p* = 0.208). As expected, both 39,X^Y*^O and 40,XY groups showed a significant increase in exploration of the familiar objects in their novel location relative to their exploration in the original location (one-tailed one-sample *t*-tests: *t*[7] = 3.437, *p* = 0.005 and *t*[7] = 2.220, *p* = 0.031 respectively), but there was no significant effect of KARYOTYPE on this measure (increase in exploration, 40,XY: 26.9 ± 12.1%, 39,X^Y*^O: 19.9 ± 5.8%, *t*[14] = 0.592, *p* = 0.563).

### 3.5 1-Choice serial reaction time task

All 12 39,X^Y*^O mice tested met stable baseline performance criteria after training, but only 6 of the initial cohort of 40,XY mice met these criteria; the 5 40,XY mice who did not failed to perform sufficient numbers of trials due to low levels of panel pushing and/or nosepoking behaviour. Overall trial number, accuracy, omissions, nosepoke, panel push and activity data for the three manipulation sessions are described in Table 1. The most striking finding was that mutant mice tended to make significantly more responses (nosepokes) than their wildtype counterparts across all three sessions, but equivalent numbers of trial initiations (panel pushes). We also noted greater overall activity in the mutant mice during testing as indexed by the number of infra-red beam breaks. The number of nosepokes performed was not directly related to the general activity of the mice as indexed by infra-red beam breaks for any of the three manipulation sessions (LITI: Spearman correlation coefficient = 0.146, *p* = 0.603, ELITI: *r* = 0.260, *p* = 0.350, I10: *r* = 0.382, *p* = 0.161).

**Figure 2:**
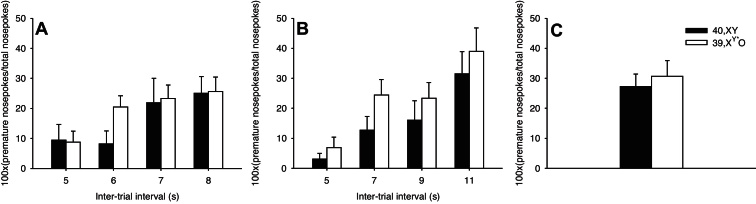
Number of premature nosepokes normalised for total nosepoking in 39,X^Y*^O (*n* = 12) and 40,XY (*n* = 6) mice for manipulation sessions in which the inter-trial interval (ITI) was pseudorandomly varied between 5 and 8 s (A) or between 5 and 11 s (B), and in which the ITI was held constant at 10 s (C).

As our main index of impulsivity in this task (premature nosepokes) could potentially be confounded by the group difference in response behaviour, we normalised for general nosepoking rates in our analysis. Whilst the pattern of data from the LITI and ELITI manipulations was as expected (i.e. more ‘normalised’ premature responses for longer ITIs, effect of ITI: *F*[3,48] = 9.538, *p* < 0.001 and *F*[3,48] = 18.935, *p* < 0.001 respectively), there was no effect of KARYOTYPE on either manipulation (*F*[1,16] = 0.329, *p* = 0.574 and *F*[1,16] = 1.051, *p* = 0.321 respectively) nor any KARYOTYPE × ITI interaction (*F*[3,48] = 1.530, *p* = 0.219 and *F*[3,48] = 0.318, *p* = 0.812 respectively) (Fig. 2A and B). In the I10 manipulation, both groups of mice tended to make high numbers of normalised premature responses as predicted for this consistently extended ITI, but again there was no significant effect of KARYOTYPE (*t*[16] = −0.432, *p* = 0.671, Fig. 2C).

**Figure 3:**
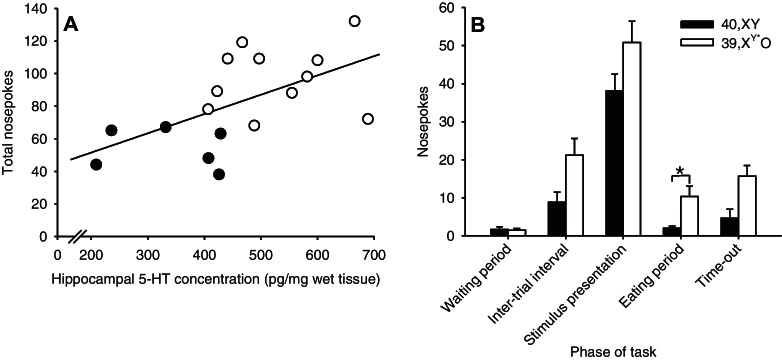
Relationship between nosepoking behaviour and hippocampal 5-HT in 39,X^Y*^O (*n* = 12, white circles) and 40,XY (*n* = 6, black circles) mice (A), and the allocation of these nosepokes across different phases of the task (B) during the ELITI manipulation session.

Hippocampal lesions have previously been shown to influence response rates in rodents in an appetitively-motivated operant task (Shull and Holloway, 1985). Hence, we tested whether the increased nosepoking behaviour seen in 39,X^Y*^O mice could be explained by their elevated hippocampal 5-HT levels. We observed significant positive correlations between hippocampal 5-HT levels and total nosepokes in the LITI and ELITI manipulation sessions (Spearman correlation coefficient = 0.571, *p* = 0.017 and *r* = 0.579, *p* = 0.015, respectively); data from the ELITI session are shown in Fig. 3A. Whilst there was a trend towards correlation between hippocampal 5-HT and total nosepokes in the I10 session this was non-significant (*r* = 0.463, *p* = 0.061). Hippocampal 5-HT levels correlated specifically with nosepoking behaviour, in that there was no relationship with total panel pushes/beam breaks across any of the three manipulation sessions (panel pushes: LITI: *r* = 0.217, *p* = 0.404, ELITI: *r* = 0.351, *p* = 0.167, I10: *r* = 0.243, *p* = 0.346, beam breaks: LITI: *r* = 0.056, *p* = 0.850, ELITI: Spearman correlation coefficient = −0.024, *p* = 0.935, I10: *r* = 0.132, *p* = 0.654).

In order to specify more precisely how nosepoking behaviour differed between 39,X^Y*^O and 40,XY mice, post hoc, we subdivided the central nosepokes made during the ELITI manipulation into five categories according to when they were made: (i) after completion of a trial but pending initiation of a subsequent trial (‘waiting period’), (ii) within the ITI (‘premature responses’), (iii) during stimulus presentation, (iv) within the 5 s ‘eating’ period following a correct response (traditionally regarded as ‘perseverative responses’ (Robbins, 2002)) or (v) within a time-out period. Unsurprisingly, given the high degree of stimulus control required to complete the task, the majority of nosepokes were made during stimulus presentation (Fig. 3B). Although 39,X^Y*^O mice tended to make more nosepokes than 40,XY mice across all five response categories, only for ‘perseverative’ nosepokes was there a significant effect of KARYOTYPE (*t*[11.8] = −3.016, *p* = 0.011, Fig. 3B); effect of KARYOTYPE for ‘waiting period’: *p* = 0.820, for premature responses: *t*[16] = −1.906, *p* = 0.075, during stimulus presentation: *t*[16] = −1.476, *p* = 0.159, for timeout: *p* = 0.067.

### 3.6 DHEA levels and correlations with hippocampal 5-HT and locomotor activity

**Figure 4:**
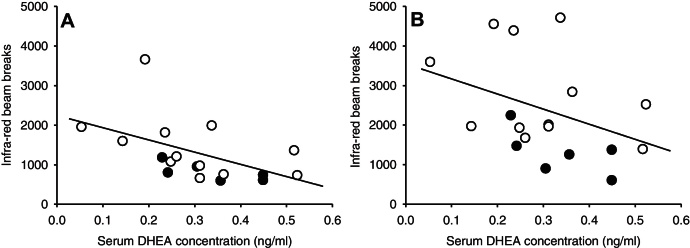
Relationship between locomotor activity (as indexed by infra-red beam breaks) and serum dehydroepiandrosterone (DHEA) levels in 39,X^Y*^O (*n* = 12, white circles) and 40,XY (*n* = 6, black circles) mice during the first hour of testing in the light (A) and during the subsequent 2 h of testing in the dark (B).

Two mice (one 39,X^Y*^O, one 40,XY) were excluded from the DHEA analysis as outliers. We observed a highly significant decrease in serum DHEA levels in 39,X^Y*^O mice relative to 40,XY mice (0.25 ± 0.04 ng/ml vs. 0.46 ± 0.06 ng/ml, *t*[27] = 3.094, *p* = 0.005). Investigating the relationship between hippocampal 5-HT levels and serum DHEA levels in mice in which both measures were assessed suggested no significant linear relationship (*r* = −0.129, *p* = 0.621, Supplementary Fig. 4). We found that 39,X^Y*^O mice were significantly more active than 40,XY mice as indexed by the total number of infra-red beam breaks during a 3-h test session (4272 ± 543 vs. 2118 ± 289, *p* = 0.003, Supplementary Fig. 5), recapitulating our previous findings (Trent et al., 2012b). There was a significant inverse correlation between total beam breaks and serum DHEA levels (Spearman correlation coefficient = −0.480, *p* = 0.044); this relationship was stronger during the first hour of the test (Spearman correlation coefficient = −0.618, *p* = 0.006, Fig. 4A) than during the remaining 2 h (Spearman correlation coefficient = −0.386, *p* = 0.113, Fig. 4B).

## 4 Discussion

Deficiency for genes within Xp22.3 may influence vulnerability to, and the symptoms of, neurodevelopmental disorders including ADHD and ASDs. We have shown here that the 39,X^Y*^O mouse, which exhibits behavioural and endocrinological features relevant to such disorders, lacks the murine orthologues of the Xp22.3 genes *STS* and *ASMT* previously implicated in psychiatric phenotypes; this finding confirms that *Asmt*, like *Sts*, is pseudoautosomal in MF1 mice, and clarifies the genetic lesion in 39,X^Y*^O mice. The main purpose of the experiments described herein was threefold: (i) to identify potential behavioural correlates of increased hippocampal 5-HT levels in 39,X^Y*^O mice using tasks known to be sensitive to hippocampal and/or serotonergic function, (ii) to test whether reduced serum DHEA levels in 39,X^Y*^O mice were systematically related to hippocampal 5-HT levels, and (iii) to investigate whether serum DHEA levels were inversely correlated with locomotor activity, as predicted by the ADHD literature.

With regard to the first aim, we found no evidence that elevated hippocampal 5-HT levels in 39,X^Y*^O mice resulted in altered ‘foraging’ behaviour or in reactivity to familiar objects in novel locations; nor did we find any evidence for an enhanced ‘ability to wait’, one aspect of impulsive function, in 39,X^Y*^O mice. The finding regarding ‘ability to wait’ contrasts somewhat with our recent 5-CSRTT data which hinted at reduced premature responding in 39,X^Y*^O mice relative to 40,XY subjects, equivalent total nosepoking across groups, and reduced nosepokes per trial (Davies et al., 2009; unpublished data). One possible explanation for this discrepancy is that our previous work examined responding across an array, and thus had a significant visuospatial attentional component; here, this component was reduced by assaying responding at just one location. We speculate, tentatively, that a reduced tendency to respond prematurely in situations where the location of the stimulus is unpredictable (i.e. in the 5-CSRTT), but a tendency to respond more frequently in predictable situations (i.e. where the stimulus location is invariant), may reflect altered decision-making in 39,X^Y*^O mice, a cognitive construct known to be sensitive to 5-HT manipulations (de Visser et al., 2011).

39,X^Y*^O mice exhibited an increased tendency to investigate the location of a previously consumed reinforcer (‘foraging’ task), and an increased tendency to respond in the 1-CSRTT, relative to 40,XY mice. The first finding may simply be a function of the greater activity of the 39,X^Y*^O mice within the arena; alternatively (or additionally), given that the mice have to actively make a choice to investigate the pot, it may reflect an elevated level of ‘checking’ behaviour, or perseveration of an unrewarded response, in these mice. The second finding, which appeared to be related to hippocampal 5-HT levels but not general activity or motivation (as indexed by panel pushing behaviour), could also be explained by increased perseveration in the mutant mice, particularly given that they made significantly more redundant nosepokes following a correct response. 39,X^Y*^O mice achieve higher scores than 40,XY mice on a progressive ratio (PR) task in which subjects must make increasingly high numbers of nosepokes in a central aperture for reinforcement (Trent et al., 2012a); as this nosepoking behaviour correlated with hippocampal 5-HT levels but not with general activity levels, we speculated previously that rather than reflecting enhanced motivation, the altered performance of the 39,X^Y*^O mice on the PR task may reflect an elevated tendency to nosepoke perseveratively (Trent et al., 2012a). Our current data support this idea, and strengthen the notion that deficiency for *Sts* and/or *Asmt* may predispose to perseverative behavioural patterns via influencing levels of hippocampal 5-HT. As *Sts* is expressed in the hippocampus and is known to influence hippocampal neurochemistry (Rhodes et al., 1997) its deficiency represents the most convincing underlying candidate mechanism. *Asmt* brain expression appears to be limited to the pineal gland (Kasahara et al., 2010); hence, any behavioural and neurochemical sequelae arising from its absence are likely to be indirect and mediated by effects on systemic melatonin levels.

The idea that elevated hippocampal 5-HT results in increased perseveration is consistent with the ‘perseveration-inducing’ effects of hippocampal lesions in rodents across a number of assays (e.g. Dalland, 1970; Maruki et al., 2001) including the 5-CSRTT (Le Pen et al., 2003), and the effects of systemically-administered drugs targeting the 5-HT system on perseveration in behavioural tasks (Brigman et al., 2010; Rhoads et al., 2012). Perseveration in serial reaction time tasks has been suggested to be a form of compulsive behaviour (Robbins, 2002). 40,XY and 39,X^Y*^O mice do not differ on a marble-burying task thought to index aspects of compulsivity (Trent et al., 2012a), and so it is likely that the neural substrates underlying marble-burying behaviour and perseverative responding in 39,X^Y*^O mice are largely dissociable. In order to establish a causal relationship between hippocampal 5-HT levels and perseverative behaviour in 39,X^Y*^O mice rather than the association suggested here, it would be worthwhile examining the behavioural effects of drugs targetting the 5-HT system infused directly into the hippocampus. It may also be useful to examine the extent to which heightened perseveration in 39,X^Y*^O mice is manifest across other paradigms taxing behavioural flexibility such as reversal learning. Prenatal administration of the teratogen valproic acid in rats, a model of autism, results in both altered hippocampal 5-HT levels and behavioural inflexibility in adulthood (Narita et al., 2002; Dufour-Rainfray et al., 2010; Bambini-Junior et al., 2011). Therefore, we speculate that, in rodents (and possibly in humans), abnormalities in hippocampal 5-HT levels giving rise to perseverative behaviour may represent a common endpoint of multiple genetic mutations or environmental insults, and may be modifiable pharmacologically. How altered hippocampal 5-HT levels might influence response rate/perseveration remains to be clarified, but it is possible that the integrity/function of neural projections from the hippocampus to the medial prefrontal cortex, a brain region regulating compulsive perseveration in rodents (Muir et al., 1996) may be compromised.

We previously speculated that reduced DHEA levels in 39,X^Y*^O mice might result in increased activity of tryptophan hydroxylase, the rate-limiting enzyme in the 5-HT biosynthetic pathway, and that this may ultimately explain elevated 5-HT levels in the hippocampus of these mice (Trent et al., 2012a). The lack of a relationship between systemic DHEA levels and hippocampal 5-HT levels here argues against this view, although it should be acknowledged that there may be a stronger relationship at other developmental timepoints.

Work in individuals diagnosed with ADHD has suggested that reduced levels of systemic DHEA may be particularly associated with hyperactive symptomatology (Strous et al., 2001). However, these studies have been difficult to interpret given possible confounding demographic and treatment variables. Here, we have shown under controlled experimental conditions that, consistent with the clinical literature, there is evidence for an inverse relationship between systemic DHEA levels and activity. The correlation between DHEA level and activity was particularly strong during the first hour of the present assay, suggesting that levels of this steroid may particularly impact upon activity within a novel environment. Again, it will be important to establish a causal relationship between DHEA levels and activity through pharmacological manipulation of the former and recording of the latter. Striatal 5-HT levels are elevated in 39,X^Y*^O mice and positively correlate with baseline activity (Trent et al., 2012a). It is feasible that DHEA levels influence this axis, and, in future work it would be interesting to test for a relationship between systemic DHEA and striatal 5-HT levels. Furthermore, striatal dopaminergic function has been implicated in locomotor hyperactivity (Mehler-Wex et al., 2006); as 39,X^Y*^O mice show a trend towards elevated dopamine levels in this brain region (Trent et al., 2012a), a link between the genetic lesion in these mice, aberrant striatal dopaminergic function, and hyperactivity might also be considered.

Theoretically, the abnormal behavioural phenotypes we have previously described in 39,X^Y*^O mice (Davies et al., 2009; Trent et al., 2012a,b) could be due to deficiency for *Sts* and/or *Asmt*. However, as pharmacological manipulation of the Sts axis influences attention and aggression (Nicolas et al., 2001; Davies et al., 2009), and given strong genetic and biochemical evidence for Sts in aggression (Mortaud et al., 2010), these two phenotypes in 39,X^Y*^O mice are likely to result specifically from the absence of *Sts*. The present data showing that locomotor activity correlates with levels of the Sts product DHEA suggests that hyperactivity in 39,X^Y*^O mice is predominantly due to loss of Sts (and its downstream endocrinological effects). It is formally possible that the activity phenotype is subtly affected by abnormalities in melatonin biosynthesis arising from loss of the *Asmt* gene (Korf et al., 2003), although it should be noted that 39,X^Y*^O mice do not show any obvious changes in photoperiod relative to 40,XY mice (Trent et al., 2012b) as might be expected if the melatonin system is severely compromised. In the future, should the production of single gene *Sts* and *Asmt* knockout rodents be possible, they could be used to unambiguously dissociate between the behavioural and neural effects of the two genes.

Our mouse data suggest that mutations or polymorphisms resulting in reduced Sts and/or Asmt function may be associated with rates of perseveration and altered hippocampal serotonergic function in neurodevelopmental disorders of behavioural inflexibility such as ADHD and autism; serotonergic abnormalities have long been implicated in the pathogenesis of autism (Scott and Deneris, 2005), and may also influence ADHD symptomatology (Oades, 2007). The current findings further suggest that genetic variants within *STS* resulting in reduced systemic DHEA levels may also be associated with hyperactivity. The 39,X^Y*^O mouse has both face and construct validity as a model for neurodevelopmental disorders: it is inattentive, hyperactive, emotionally hyper-reactive, aggressive and behaviourally inflexible, as a consequence of lacking genes previously implicated in ADHD and autism pathology. Further work in this mouse should enable us to further elucidate the molecular, cellular, and biochemical underpinnings of its abnormal behavioural and neural phenotypes.

## Role of the funding sources

The funding bodies had no role in study design; in the collection, analysis and interpretation of data; in the writing of the report; and in the decision to submit the paper for publication.

## Conflict of interest

All authors declare that they have no conflicts of interest.

## Figures and Tables

**Table 1:** Data from the three 1-CSRTT manipulation sessions for 40,XY (*n* = 6) and 39,X^Y*^O mice (*n* = 12).

Manipulation	Karyotype	Trial number	% Accuracy	% Omissions	Nosepokes	Panel pushes	Beam breaks†
LITI	40,XY	51 ± 2	94 ± 3	41 ± 14	42 ± 9	157 ± 28	721 ± 292
	39,X^Y*^O	70 ± 5**	96 ± 1	9 ± 2	98 ± 4**	172 ± 22	863 ± 140

ELITI	40,XY	50 ± 4	97 ± 1	23 ± 2	54 ± 5	146 ± 26	537 ± 51
	39,X^Y*^O	59 ± 5	96 ± 1	14 ± 3*	99 ± 6**	173 ± 18	905 ± 146*

I10	40,XY	38 ± 7	93 ± 5	24 ± 7	45 ± 9	118 ± 30	465 ± 106
	39,X^Y*^O	46 ± 5	97 ± 1	13 ± 4	87 ± 8**	149 ± 17	912 ± 124*

* *p* < 0.05.

** *p* < 0.01.

† Activity data was not collected for one 40,XY mouse and two 39,X^Y*^O mice due to technical problems with the infra-red beams.
