# Supplementary material for: Biological mechanisms associated with increased perseveration and hyperactivity in a genetic mouse model of neurodevelopmental disorder
Source: Psychoneuroendocrinology. 2013 Aug;38(8):1370–80. doi: 10.1016/j.psyneuen.2012.12.002 (PMC3690523; doi:10.1016/j.psyneuen.2012.12.002)
Supplement: Supplementary file 1 [file mmc1.doc]

**Biological mechanisms associated with increased perseveration and hyperactivity in a genetic mouse model of neurodevelopmental disorder**

Simon Trent^1,2^, Rachel Dean^3^, Bonnie Veit^3^, Tommaso Cassano^4^, Gaurav Bedse^5^, Obah A. Ojarikre^6^, Trevor Humby^1,2^, William Davies^1,2^*

^1^Behavioural Genetics Group and Neuroscience and Mental Health Research Institute, Schools of Psychology and Medicine, Cardiff University, Cardiff, UK

^2^Institute of Psychological Medicine and Clinical Neurosciences and MRC Centre for Neuropsychiatric Genetics and Genomics, School of Medicine, Cardiff University, Cardiff, UK

^3^School of Psychology, Cardiff University, Cardiff, UK

^4^Department of Clinical and Experimental Medicine, Medical School, University of Foggia, Italy

^5^Department of Physiology and Pharmacology, Sapienza University of Rome, Rome, Italy

^6^Division of Stem Cell Biology and Developmental Genetics, MRC National Institute for Medical Research, London, UK

**Supplementary Material**

This material supplements but does not replace the content of the peer-reviewed paper published in Psychoneuroendocrinology.

*‘Foraging’ task detailed experimental methods*

During three sessions of initial shaping (one session of three trials per day), mice were trained to obtain a drop of reinforcer from white ceramic containers (7cm diameter, 4cm height) placed at one end of a plastic testcage (45x12.5x11.5cm, lxbxh). Only when an animal had consumed one drop of the reinforcer and revisited the pot once, was a second drop was added; this protocol ensured that animals were aware that once the reinforcer had been obtained, it would not be replenished. For session one, the ceramic container was placed on the floor for all three trials, for session two, it was elevated 4cm on a wooden block on trials 2 and 3 (forcing the mouse to make a discrete consummatory choice), and for session three, it was elevated for all three trials. Mice were then extensively habituated to the test arena (white perspex box, 98x98x30cm, lxbxh, illuminated from above at 220 lux, the minimum light level necessary for adequate video-recording) for three sessions (one session per day); during session one of habituation, mice were placed in the centre of the arena and allowed to explore freely for 10mins. During this ‘open field’ session, the number of times a mouse entered a different quadrant was recorded as an index of activity. On sessions two and three of habituation, two elevated ceramic containers each containing one drop of reinforcer were introduced to the arena; exploration was allowed to continue until both drops had been consumed, or for a maximum of 10mins. Mice were then given five consecutive test sessions (one per day), before which eight elevated containers, each holding one drop of reinforcer, were arranged in the arena in a manner designed to restrict systematic searching and to be unaversive (**Supplementary Figure 1**). Test sessions lasted until all reinforcers had been obtained, or for a maximum of 15mins. The arena and containers were thoroughly cleaned with 1% acetic acid between mice to remove odour cues. Main measures of interest at baseline performance (i.e. test day 5) included the number of reinforcers obtained within the time limit, the latency to obtain these reinforcers, and the number of errors made in obtaining the reinforcers (i.e. repeat visits to previously visited containers). On the following day, a single probe session was then run in which extra-maze cues were excluded using a curtain around the test arena to determine whether the two experimental groups were using extra-maze cues differentially. After a further standard test session equivalent to test day 5 the next day, a second probe session was performed on the final day of testing (day 14) whereby only one container was placed in the arena, and animals were free to explore for 10mins; the latency to consume the reinforcer, and number of repeat visits to the container once the reinforcer had been consumed (a potential index of perseveration) were recorded.

**Supplementary Figure 1:** Plan view of the arrangement of pots for the ‘foraging’ spatial navigation task during habituation (a), during the test proper (b), and during the one-pot variant (c). Given previous evidence for elevated anxiety-related behaviours in 39,X^Y*^O mice (Trent et al., 2012), no pots were placed in the most aversive central portion of the open field. Pots were not arranged in a simple circle in an attempt to negate systematic searching strategies.

*Object-location task detailed experimental methods*

The task was run in the same arena as the foraging task, illuminated at 220 lux. All sessions were videotaped, and most data analysis was done *post hoc*. The mice were habituated to the arena for three sessions (one session per day, each of 10mins duration). For sessions one and two the arena contained no objects; for session three it contained four selected novel objects (**Supplementary Figure 2**) placed in a square 10cm apart. There were then two test days, with two days break between each. Each test day consisted of a sample phase and a test phase. Initially, a 40,XY mouse was run, with a sample phase of 10mins during which the total time spent investigating the objects (defined as an animal directing its nose toward an object at a distance of <2cm, or touching an object with its nose) was recorded; the mouse was then returned to its homecage for 2mins, and the arena and objects were cleaned with 1% acetic acid to remove odour cues. The two diagonally opposite objects were switched during this interval. The mouse was then reintroduced for a 10min test phase. A ‘partner’ 39,X^Y*^O mouse was then given sample and test phases, with the sample phase lasting until the 39,X^Y*^O mouse’s total exploration time was identical to that of its 40,XY equivalent; the test phase for the 39,X^Y*^O mouse was always of 10mins duration. The above procedure was repeated for all eight pairings on test day 1. On test day 2, the opposite pair of objects were diagonally switched. Switched objects, and their locations, were pseudorandomised across mice and test days. The testing procedure is summarised in **Supplementary Figure 3**. Data were averaged across test days one and two. Exploration time for each object in the sample and test phases was recorded. From this, the ‘total percentage exploration time for the ‘objects to be swapped’’ in the sample phase, and the ‘total percentage exploration time for ‘swapped objects’’ in the test phase was calculated, and thereafter the main measure of ‘percentage change in exploration of these objects across sample and test phases’. This analysis strategy normalised for inter-individual differences in absolute levels of exploration, eliminated any systematic experimenter bias, and mitigated against skewed data arising from the anomalous exploration of any one object.

**Supplementary Figure 2:** Objects for the object-location task**.** The objects used had previously been used in studies of hippocampal function at Cardiff University (Good et al., 2007); importantly, they were of no natural significance to the mice, were sturdy, non-porous, matched for size and shape, difficult to climb, and had not previously been associated with reinforcement.

**Supplementary Figure 3:** summary of protocol for the object-location task**.** During the test phase, the position of two diagonally opposite objects was switched. The main measure of interest was the % increase in relative exploration of the ‘swapped’ objects in the test phase relative to the extent to which they were explored in the sample phase.

**Supplementary Figure 4.** Relationship between serum DHEA levels and hippocampal 5-HT levels in 39,X^Y*^O (n=12, white circles) and 40,XY (n=6, dark circles) mice

**Supplementary Figure 5.** Locomotor activity in 39,X^Y*^O and 40,XY mice as indexed by infra-red beam breaks in an activity cage over a 3hr (180min) period (lights off after 60mins).

**References**

Trent, S., Dennehy, A., Richardson, H., Ojarikre, O. A., Burgoyne, P. S., Humby, T., Davies, W., 2012. Steroid sulfatase-deficient mice exhibit endophenotypes relevant to attention deficit hyperactivity disorder. Psychoneuroendocrinology. 37, 221-229.

Good, M. A., Barnes, P., Staal, V., McGregor, A., Honey, R. C., 2007. Context- but not familiarity-dependent forms of object recognition are impaired following excitotoxic hippocampal lesions in rats. Behav Neurosci. 121, 218-223.
